# Flavor, relative palatability and components of cow’s milk hydrolysed formulas and amino acid-based formula

**DOI:** 10.1186/s13052-015-0141-7

**Published:** 2015-06-03

**Authors:** Michele Miraglia Del Giudice, Enza D’Auria, Diego Peroni, Samuele Palazzo, Giovanni Radaelli, Pasquale Comberiati, Francesca Galdo, Nunzia Maiello, Enrica Riva

**Affiliations:** Department of Pediatrics, II University of Naples, via De Crecchio 4, 80138 Napoli, Italy; Department of Pediatrics, San Paolo Hospital-University of Milan, Via A di Rudinì 8, I-20142 Milan, Italy; Department of Pediatrics, University of Verona, Policlinico Borgo, Roma, Verona Italy

**Keywords:** Cow’s milk hydrolysed formulas, Amino acid-based formula, Palatability

## Abstract

**Background:**

Both extensively hydrolysed formulas (eHF) and amino acid-based formula (AAFs) have been demonstrated effective for the treatment of CMA. However, in clinical practice, parents complain that hydrolysates are rejected by children due to their bad taste. Flavor of hydrolysed formulas has been poorly investigated although it affects the acceptance of milk over all the other attributes. The aim of the present study was to understand the factors underlying the unpleasant flavor of hydrolysed 25 formulas and amino acid-based formula.

**Subjects and methods:**

One hundred and fifty trained panelists performed a randomized-double-blind test with different milks. The smell, texture, taste and aftertaste of each formula were evaluated on a scale ranging from −2 (worst) to 2 (best).

**Results:**

Formulas showed significant difference, as compared to cow’s milk, in smell, texture, taste and aftertaste. Overall, whey eHFs were judged of better palatability than casein eHF and the AAFs (p < 0.05). Whey eHF showed significant differences among them for sensory attributes, especially for taste and aftertaste.

**Conclusions:**

These results suggest that a broad range of flavor exists among the hydrolysed formulas. Further studies, adequately designed to investigate the relationship between milks’ flavor and nutrient profile of hydrolysed formulas are warranted.

## Introduction

Cow’s milk allergy (CMA) affects 2-6% of children with a prevalence peak during the first year of age [1]. The prognosis of CMA is good with a recovery of about 50% at one year and 80% by the fifth year of age [2,3], although recent studies suggest that CMA may now be a more persistent disease [4-6]. The effective treatment for CMA is cow’s milk protein exclusion diet.

The formulas intended for treatment (e.g. extensively hydrolysed formulas) should contain only peptides with molecular weight <3000 Daltons [7]. Extensively hydrolysed formulas (eHF) are comprised of short peptides mostly < 1500 dalton and free amino acids obtained by enzymatic hydrolysis followed by further processing such as heat treatment and/or ultrafiltration.

Although the majority of infants and children with CMA tolerate an extensively hydrolyzed formula with whey or casein as a nitrogen source, some infants may react to eHF as they possess residual allergenicity.

Indeed, It has been recommended that dietary products for treatment of CMA in infants should be tolerated by at least 90% (with 95% confidence) of infants with CMA in clinical studies [8]. Only some extensively hydrolysed formulas (eHF) and amino acid-based formula (AAF), that are considered anallergenic, meet these criteria [9].

Current Guidelines recommend to use a milk-based extensively hydrolysed (eHF) formula as the first choice for the treatment of CMA [10-12]. The use of amino acid-based formula is recommended in infants with CMA characterized by severe symptoms and in infants not tolerating eHF [13,14].

Aminoacid based formula should be used as first line-treatment in the case of anaphylaxis and eosinophilic esopaghitis [12,15]. Moreover, they may be considered as first therapeutic option in infants with multiple food allergies or in the presence of severe enteropathy indicated by hypoproteinemia and failure to thrive [11,16,17].

Both EHF and AAF have been demonstrated effective for the treatment of CMA. However, in clinical practice, frequent complaints by parents are that hydrolysates are rejected by children due to the bad taste [18,19].

Thus an understanding of the factors that underlie their unpleasant flavor is important and could have the practical result of improving their palatability thereby enhancing acceptance.

The unpleasant flavor of hydrolysed has been related to the peptides produced during proteolysis [20]. Other authors postulate that the taste depends on the enzymes used in proteolysis [21].

Pedrosa et al. found a correlation between the amount of bitter peptides and the palatability of formulas [22].

However, apart from bitterness, we hypothesize that other sensory attributes may contribute to the palatability of hydrolysed formulas.

The aim of the present study was to understand the factors underlying the unpleasant flavor of hydrolysed formulas.

## Materials and methods

This double-blind multi-centre study was carried out in healthy young adults to assess the flavor and relative palatability of different cow’s milk protein hydrolysed formulas and amino acid-based formula. One hundred and fifty subjects (males 71, females 79; mean (SD) age 28.3 (3.9) years (range 25–30) were recruited among the staff of three Italian University Hospitals (fifty for each centre: San Paolo Hospital, Milan; Department of Pediatrics, Second University of Naples and Department of Pediatrics, University of Verona). Inclusion criteria were: age < 30 years, being non-smokers, willingness to participate. Subjects suffering from rhino-sinusal acute and any acute and chronic diseases were excluded. At recruitment the subjects received detailed explanations about the aim of the study and signed a consent form. The panelists were trained in two 1-h sessions on each product tasted.

The local Hospital Ethics Committee approved the study protocol. The assessed milks samples included whole cow’s milk (reference), a starting formula, a partially hydrolysed formula, five whey protein extensive hydrolysed formulas, a casein extensive hydrolysed formula and two amino acid-based formulas. The formulas were selected among those currently available on the market and were representative of whey or casein based-hydrolysed formulas and amino acid mixtures.

Energy and macronutrients composition of whole cow’s milk, starting formula, cow’s milk protein hydrolysed formulas and amino-acid based formulas, as provided by the manufacturers, are shown in Table 1.

**Table 1:** Energy and macronutrients composition of cow’s milk, follow on formula, pHF (partial idrolized formula), whey eHF (whey protein extensive hydrolized formulas), casein eHF (casein extensive hydrolized formula), AABF (amino acid mixture)*

***Milk***	***Energy Kcal/100 ml***	***Proteins g/100 ml***	***Lipids g/100 ml***	***Nucleotides mg/100 ml***	***Carbohydrates, g/100 ml***
***Cow’s milk***	*66*	*3.2*	*3.9*		*4.8*
			*S:2.4*		*LA:4.8*
			*M:1.1*		
			*P:0.1*		
***Follow on formula***	*67*	*1.2*	*3.6*	*2*	*7.5*
		*Cs/S:30/70*	*L:0.57*		*LA:7.5*
			*LN:0.068*		
***pHF***	*67*	*1.3*	*3.4*	*2*	*7.8*
		*Cs/S:0/100*	*S:1.4*		*LA:7.8*
		*Peptides:*	*M:1.2*		
		*<600:18.8*	*P:0.6*		
		*600-3000:63.2*	*L:0.524*		
		*3000-5000:11.8*	*LN:0.064*		
		*>5000:6.1*			
***Whey eHF 1***	*72*	*2.1*	*3.6*	*6*	*7.7*
		*Cs/S:0/100*	*S:1.75*		*DTM:6.7*
		*Peptides:*	*M:0.99*		
		*<240:16.9*	*P:0.6*		
		*360-600:44.6*	*MCT:1.44*		
		*600-1200:30.2*	*L:0.51*		
		*1200-2400:7.1*	*LN:0.064*		
		*2400-4000:1.2*			
***Whey eHF 2***	*67*	*1.65*	*3.43*	*0*	*7.33*
		*Cs/S:0/100*	*S:1.33*		*DTM:3.5*
		*Peptides:*	*M:1.23*		*LA:3.83*
		*<240:16.9*	*P:0.62*		
		*360-600:44.6*	*MCT:1.37*		
		*600-1200:30.2*	*L:0.5*		
		*1200-2400:7.1*	*LN:0.05*		
		*2400-4000:1.2*			
***Whey eHF 3***	*62.5*	*1.6*	*3.4*	*0*	*6.5*
		*Cs/S:0/100*	*S:1.24*		*DTM:3.4*
		*Peptides:*	*M:1.55*		*LA: <0.12*
		*0-100:5.4*	*P:0.61*		
		*100-500:45.2*	*L:0.44*		
		*500-1000:26.7*	*LN:0.05*		
		*1000-1500:10.8*			
		*1500-3000:9.1*			
		*3000-4000:2*			
		*>4000:0.8*			
***Whey eHF 4***	*66*	*1.8*	*3.5*	*3.22*	*6.8*
		*Cs/S:0/100*	*S:2.1*		*PO: 5.7*
		*Peptides:*	*M:0.8*		
		*<200:15.1*	*P:0.6*		
		*200-400:28.8*	*MCT:1.8*		
		*500-1500:31.8*	*L:0.476*		
		*1500-2500:14.3*	*LN:0.088*		
		*2500-3500:6.7*			
		*3500-4500:2.5*			
		*>4500:0.8*			
***Whey eHF 5***	*66*	*1.6*	*3.5*	*3.22*	*7.1*
		*CS/S:0/100*	*S:1.5*		*LA:2.9*
		*<200:15.1*	*M:1.4*		*PO: 3.6*
		*200-400:28.8*	*P:0.6*		
		*500-1500:31.8*	*MCT:1.08*		
		*1500-*	*L:0.456*		
		*2500:14.3*	*LN:0.084*		
		*2500-3500:6.7*			
		*3500-4500:2.5*			
		*>4500:0.8*			
***Casein eHF 1***	*68*	*1.9*	*3.4*	*0*	*7.5*
		*Cs/S:100/0*	*S:1.45*		
		*Peptides:*	*M:1.26*		
		*<500:60*	*P:0.67*		
		*500-1000:35*	*MCT:0.09*		
		*1000-2000:4*	*L:0.61*		
			*LN:0.046*		
***AABF 1***	*67*	*1.8*	*3.4*	*0*	*7.2*
			*S:1.2*		
			*M:1.3*		
			*P:0.66*		
			*MCT:0.136*		
			*L:0.579*		
			*LN:0.0579*		
***AABF 2***	*68*	*1.89*	*3.6*	*0*	*7*
			*S:1.54*		
			*M:1.33*		
			*P:0.71*		
			*MCT:0.1*		
			*L:0.58*		
			*LN:0.054*		

* Cs/S:casein/serum protein; PM:%; Satured (S); Monoinsatured (M); Polynsatured (P); Medium chain tryglicerides (MCT); Linoleic acid (L;) Linolenic acid (LN); dextrin maltose (DTM); polisaccardies (PO); lactose (LA).

Total energy ranged from 62.5 Kcal/100 ml (whey eHF 3) to 72 Kcal/100 ml (whey protein hydrolysed ) and protein from 1.6 g/100 ml (whey protein EHF 3 and 5) to 2.1 g/100 ml (whey protein hydrolysate 1). The total fats’ amount of the tested formulas was comparable with that of cow’s milk and starting formula both, while protein and CHO were lower and higher than cow’s milk, respectively (p < 0.001).

Extensively whey hydrolysates 1,2,3 and casein hydrolysed formula contained more than 50% of peptides with MW lower than 500–600 Dalton (D); in particular, the casein eHF 1 had 60% of peptides with MW of 500 D and 95% lower than 1000 D.

The total fats’ content varied from 3.4 g/100 ml (whey EHF 3, casein eHF and amino acid mixture 1) to 3.6 g/100 ml (whey protein hydrolyzate 1, amino acid mixture 2). The distribution of satured, monunsatured and polyunsatured fatty acids are different among the formulas, ranging from 1.2 to 2.1 g/100; from 0.8 to 1.55 g/100 ml and from 0.6 to 0.7 g/100 ml respectively, compared to 2.4, 1.1 and 0.1 gr/100 ml of cow’s milk. Medium-chain fatty acids (MCT) were absent in partial and whey-protein hydrolysate 3; in the other formulas MCT content ranges from 0.09 g/100 ml (casein-hydrolysate) to 1.8 g/100 ml (whey protein hydrolysate 4).

All formulas contain essential long chain polyunsaturated linoleic acid (range: 0.44 to 0.61 g/100 ml) and α-linolenic acid (range: 0.046 - 0.088 g/100 ml).

The carbohydrates (CHO) content of hydrolysates varied from 6.5 g/100 ml (whey EHF 3) to 7.7 g/100 ml (whey eHF 1). In pHF total CHO content was represented by lactose. Among eHFs, lactose was contained in eHF 2.3 and 5 only.

Samples of cow’s milk and formulas were bottled in identical coded pots.

Each subject received 10 ml of cow’s milk or formula according to a within-subject randomization computer generated sequence, to eliminate order effect. The sensory tests were carried out on two days during an afternoon session at ten-minute interval.

All the subjects involved in the study were unaware of the milks administered until codes were broken after the completion of the data analysis. The same trained health worker (one per centre) distributed samples to the panelists. Milks were offered at room temperature (18-22°C). Water and unsalted crackers were used as palate cleansers between samples.

Subjects were firstly invited to smell and to take a sip of the different milks. The following attributes have been considered: smell, texture, taste and aftertaste. The panelists were asked to evaluate each attribute on a five-point scale ranging from-2 (worst) to 2 (best) (Table 2).

**Table 2:** **Definitions and scales for sensory properties used to describe formulas’ attributes**

**Attribute**	**Definition and scales**
Smell	unpleasant (−) → pleasant (+)
Texture	rough (−) → smooth (+)
Taste	unpleasant (−) → pleasant (+)
Aftertaste	long (−) → short (+)

Smell: the characteristic perceived by the olfactory sense.

unpleasant (−) → pleasant (+).

Texture: degree to which the surface of the samples feels smooth or rough in the mouth.

Rough (−) → smooth (+).

taste: the flavor perceived by the mouth.

Low (−) → High (+).

Aftertaste: length of time after swallowing the sample the flavor persists in the mouth.

Long (−) → short (+).

An overall judgement of palatability was reported by each panelist on a ten-point visual analogic scale ranging from 0 (highly negative) to 9 (highly positive) [23].

### Statistical analysis and methods

Descriptive data are reported as medians with 25th to 75th centile (Table 3). As sensory attributes were not normally distributed (Kolmogorov-Smirnov test), significant difference for each attribute among the different formulas was assessed by the Friedman test, and significance of *post-hoc* multiple comparisons by the Schaich-Hamerle and Conover tests [24]. The association of palatability with nutrient features of milks was tested by Spearman’s correlation coefficient. A significance level of 0.05 was assumed and statistical tests are two-tailed.

**Table 3:** **Flavor of the different formulas**

**Attribute** *	**Cow’s milk**	**Starting formula**	**Partial hydrolisate**	**Serum protein hydr 1**	**Serum protein hydr 2**	**Serum protein hydr 3**	**Serum protein hydr 4**	**Serum protein hydr 5**	**Casein extensive hydr**	**Amino acid mixtures 1**	**Amino acid mixtures 2**	**P value** ^**†**^
Smell	2 (1 ; 2)^**^	0 (−1 ; 1)^b^	1 (0 ; 1)^b^	0 (−1 ; 1)^b^	0 (−1 ; 1)^b^	0 (−1 ; 1)^b^	0 (−1 ; 1)^b^	0 (−1 ; 1)^b^	−2 (−2 ; −1)^c^	−1 (−2 ; −1)^c^	−2 (−2 ; −1)^c^	<0.0001
Texture	1 (0 ; 1)^a^	1 (0 ; 1)^a^	1 (0 ; 2)^a^	1 (0 ; 1)^a^	1 (0 ; 1)^a^	0 (−1 ; 1)^a^	1 (0 ; 2)^b^	1 (0 ; 1)^a^	0 (−1 ; 1)^c^	1 (0 ; 1)^a^	1 (0 ; 1)^a^	<0.0001
Taste	2 (1 ; 2)^a^	1 (0 ; 2)^b^	1 (0 ; 1)^b^	−1 (−2 ; 0)^d^	−1 (−2 ; 0)^d^	−1 (−2 ; 0)^d^	−1 (−2 ; 1)^c^	0 (−1 ; 1)^c^	−2 (−2 ; −1)^e^	−1 (−2 ; −1)^d^	−2 (−2 ; −1)^e^	<0.0001
Aftertaste	2 (1 ; 2)^a^	0 (0 ; 1)^b^	0 (−1 ; 1)^b^	−1 (−2 ; −1)^c^	−1 (−2 ; 0)^c^	−1 (−2 ; 0)^c^	0 (−1 ; 1)^b^	0 (−1 ; 1)^b^	−2 (−2 ; −1)^d^	−2 (−2 ; −1)^d^	−2 (−2 ; −1)^d^	<0.0001
Total Judgement	7.5 (6.2 ; 8.4)^a^	6.0 (5.3 ; 6.9)^b^	4.6 (3.7 ; 5.8)^b^	2.4 (1.6 ; 3.2)^c^	2.8 (2 ; 3.7)^d^	2.4 (1.3 ; 3.8)^c^	3.6 (2.8 ; 4.5)^e^	4 (3.2 ; 4.7)^e^	1.2 (0.5 ; 2.3)^f^	1.4 (0.7 ; 2.8)^f^	1.1 (0.5 ; 1.9)^f^	<0.0001

*The panelists evaluated each attribute on a scale of −2 to +2; values are medians with 25th to 75th centile quartile point.

**For each specific attribute, different row-superscripts (from a to f) indicate significant difference between formulas.

^†^Significance of the overall difference among formulas (Friedman's test).

The SPSS software, version 19.0 (SPSS Inc., Chicago. IL), was used for the statistical analysis.

## Results

**Figure 1:**
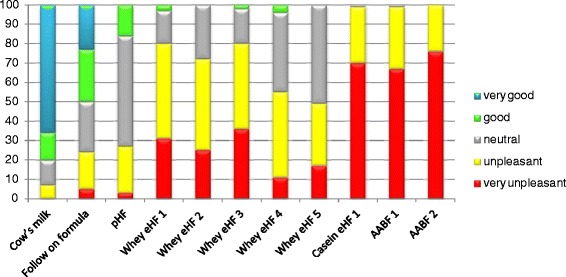
Stacked bar chart of the overall judgement of palatability of the formulas.

The scores for each sensory attribute and the overall judgement of palatability are reported in Table 3. All formulas exhibited worst overall palatability judgement than cow’s milk (p < 0.0001). Figure 1 shows the distribution of the overall judgement of palatability for cow’s milk and all the other formulas, categorized into five classes.

Formulas showed statistically significant difference, as compared to cow’s milk, in smell (0.01 p < 0.05), taste (0.0001 < p < 0.05) and aftertaste( p <0.001). Texture was different from cow’s milk for hydrolysate 3 (p < 0.05) and casein-hydrolysate formula (p < 0.05)

PHF showed better overall judgement of palatability than any eHF or AAFs (p < 0.05).

With regard to each attribute, whey-eHFs were judged as having better smell than both casein and AABFs (p < 0.05). Whey eHF3 and casein hydrolysed formula showed a rougher texture than other formulas (maximum p < 0.05). Casein hydrolysed formula and the amino acid based formula 2 were judged having the worst taste of all the formulas (maximum p < 0.05).

The aftertaste, meaning the time of persistence of unpleasant taste in the mouth, turned instead shorter for whey eHFs than for casein hydrolysed formula and AAFs (p < 0.01). Overall, whey eHFs were judged of better palatability than casein eHF and the AAFs (p < 0.05. Moreover, whey eHF showed significant differences among them for sensory attributes, especially for taste, that resulted better for the whey eHF 5 and for the aftertaste that resulted shorter for the whey eHF 4 and 5 than all other whey eHFs (p < 0.05).

The overall judgement of palatability decreased with increasing levels of total poli-unsatured fatty acids (Spearman’s correlation coefficient. 0.791, p = 0.007) and maltodextrines (Spearman’s coeff. corr. 0.723; p = 0.028), while improved with increasing levels of saturated fatty acids (Spearman’s coeff. corr. 0.640; p = 0.046), alfa-linoleic acid (Spearman’s coeff. corr. 0.740; p = 0.010) and lactose (Spearman’s coeff. corr. 0.715; p = 0.039).

## Discussion

The choice of the substitute formula for an individual child with cow’s milk allergy should be based on patients characteristics (age and symptoms), and formula’s properties, firstly documented hypoallergenicity and nutritional adequacy [25,26].

However, it is important to also consider formulas acceptance that interfere with compliance in clinical practice.

In this regard, palatability of hydrolysed formulas has been poorly investigated. This study focused primarily on the flavor and the relative palatability of hydrolysed formula and aminoacid based formulas. Moreover, it aimed also to investigate the correlation between hydrolysed formulas constituents, other than peptides, and palatability.

Our results showed significant differences of flavor of the different extensively hydrolysed formulas, with casein or whey as a nitrogen source and between eHF and amino acid based formulas. Overall, whey eHFs were judged of better palatability than casein eHF and the AAFs (p < 0.05).

It is noteworthy that whey eHF have been judged different among them for sensory attributes and overall palatability judgement. The sensory attributes that most influenced the overall judgement of palatability were taste and aftertaste, respectively.

The results also suggest that palatability improved with the increasing levels of lactose and alfa linolenic content. Concerning lactose, from a nutritional standpoint, it has several beneficial effects. Metabolic studies employing isotopic techniques in humans showed that the presence of lactose enhances the absorption and the retention of the calcium [27] and other minerals, such as magnesium and zinc [28].

Lactose naturally influences the intestinal microflora as it selectively promotes the development of putative beneficial bacteria population in the lower part of the gut [29]. In spite of the above mentioned several and well-known lactose beneficial effects, lactose continues to be excluded from the majority of the cow’s milk based-hydrolysed formulas.

Adverse reactions to lactose in cow’s milk allergy are not supported in the literature, and complete avoidance of lactose in CMA is no longer warranted. EhF containing purified lactose are now available and have been found safe and effective in the treatment of CMA [30,31].

With regard to the linolenic acid, our results agree with data on animals models that demonstrated that linoleic solutions are preferred over oleic acid and linolenic acid is preferred over linoleic acid [32].

The physiological and nutritional implications of fat sensing include gastric lipase secretion, modulated the gastro-intestinal transit, pancreatic exocrine secretions, gut hormone release, mobilization of stored lipid from enterocytes, pancreatic endocrine secretion and altered lipoprotein lipase activity [33]. Through the above activities, oral fat exposure may influence appetitive responses, food intake, nutritional status and disease risk.

Therefore, fatty acids content in special formulas needs further investigation for the all the above mentioned aspects.

The novelty of our study was to investigate the correlation between hydrolysed formulas constituents, other than peptides, and palatability.

The strenght of this study is the large number of panelists (>50) to get statistically valid data [34].

The main limitation of this study is that it was performed in young adults.

Although there are differences in taste preference between infants and adults [35], a number of studies have reported that human infants are able to discriminate different taste qualities from birth and they respond to the stimuli, especially sweet and bitter with a pattern of responses similar to those seen in adults [36].

The preference of infants and young children for sweet and aversion for bitter and sour is similar to that of most occidental adults [37].

The main differences between adults and infants are the higher preference for sweet-tasting ( meaning that infants generally prefer higher concentrations of sweet solutions than adults) and greater aversion for bitter [38,39], partly due to genetic variations and cultural differences [40].

However, it is important to point out that as preferences for taste stimuli is generally more influenced by innate factors, preferences for flavor compounds recognized by the sense of smell is more highly influenced by learning, especially early in life [41].

Experimental studies by Mennella [42] demonstrated that infants exposed before 4 month of age to hydrolysed formulas, characterized by a bitter tastes and unpleasant odor volatiles , were more wiling to accept them than older infants and the acceptance pattern that infants develop is specific to the flavor profile experienced in the first months of age [43].

However, considering that feeding with hydrolysed formulas start not infrequently after four months of age, it is as much as real that the poor palatability of hydrolysed formulas continues to be a cause of poor compliance in clinical practice [18].

Another limitation was that the ingredients of the formulas have not been independently manipulated.

Within these limitations, this study highlights that a broad range of flavor exists among the hydrolysed formulas, which seems to depend no only on the peptides content and molecular weight but also on other constituents, e.g. lipids and lactose content.

## Conclusions

In conclusion, the present study provide some useful informations on flavor of different hydrolysed formulas and on their relative palatability. However, the design of the study can not make definitive conclusions about what factors underlie these differences.

Further studies to investigate the correlation among hydrolysed formula constituents, independently manipulated, and palatability are warranted.
